# Ectopic expression of the phosphomimic mutant version of Arabidopsis response regulator 1 promotes a constitutive cytokinin response phenotype

**DOI:** 10.1186/1471-2229-14-28

**Published:** 2014-01-14

**Authors:** Jasmina Kurepa, Yan Li, Sharyn E Perry, Jan A Smalle

**Affiliations:** 1 The Rockefeller University, 1230 York Avenue, New York, NY 10065, USA; 2 Plant Physiology, Biochemistry, Molecular Biology Program, Department of Plant and Soil Sciences, University of Kentucky, 1401 University Drive, Lexington, KY 40546, USA

**Keywords:** Arabidopsis, Cytokinin signaling, Type-B response regulator, Phosphomimic mutation, Constitutive hormone response

## Abstract

**Background:**

Cytokinins control numerous plant developmental processes, including meristem formation and activity, nutrient distribution, senescence timing and responses to both the abiotic and biotic environments. Cytokinin signaling leads to the activation of type-B response regulators (RRBs), Myb-like transcription factors that are activated by the phosphorylation of a conserved aspartate residue in their response receiver domain. Consistent with this, overexpression of RRBs does not substantially alter plant development, but instead leads to cytokinin hypersensitivity.

**Results:**

Here we present comparative analysis of plants overexpressing Arabidopsis RRB 1 (ARR1) or a phosphomimic ARR1^D94E^ mutant in which the conserved aspartate-94 (D94) is replaced by the phosphomimic residue glutamate (E). The D94E substitution causes a 100-fold increase in response activation and instigates developmental and physiological changes that characterize wild-type plants treated with cytokinins or transgenic plants with increased cytokinin content.

**Conclusion:**

The current model of cytokinin signaling emphasizes the essential role of conserved aspartate residue phosphorylation of RRBs in promoting cytokinin responses. Our comparative analyses of developmental and physiological traits of ARR1 and ARR1^D94E^ overexpressing plants revealed that the ARR1^D94E^ protein is indeed a constitutive and wide-spectrum cytokinin response activator.

## Background

Cytokinins are a class of hormones that play essential roles in plant development and plant responses to the environment [1-5]. The cytokinin response pathway resembles two-component signaling mechanisms from yeast and bacteria [6]. The core cytokinin signaling pathway in Arabidopsis involves the actions of four components: the histidine kinases (CHKs), histidine phosphotransfer proteins (HPTs), and two antagonistically acting classes of response regulators (RRs) that control the gene expression outputs of the pathway [6-10]. The signaling cascade starts with cytokinin binding to a CHK receptor, resulting in its autophosphorylation at a conserved histidine [6,11-14]. The phosphate group is then transferred to a conserved aspartate residue of the receptor’s receiver domain, and then from the receiver domain to a histidine residue in a HPT [6,15-17]. The phosphorylated HPT relays the phosphate to an aspartate residue in the receiver domain of type-B RR (RRB). Phosphorylation of RRBs is thought to activate them and promote cytokinin action by up-regulating the expression of cytokinin response genes [6,18,19]. One class of transcriptional targets of activated RRBs are type-A *RR* (*RRA*) genes [20-23]. RRAs are also phosphorylated by HPTs, which increases their activity and, at least for some members, decreases their degradation rate [24]. Since RRAs act as cytokinin response inhibitors, their cytokinin-induced transcription combined with their cytokinin-dependent activation and stabilization leads to suppression of cytokinin action, thus limiting the strength and the duration of the cytokinin response [20,25,26].

In addition to the core signaling components, the cytokinin response pathway in Arabidopsis involves other positive and negative regulators. For example, Arabidopsis HPT6 (AHP6) is similar in sequence to other HPTs, but lacks the conserved histidine needed for the phosphorelay. As a result, AHP6 acts as an inhibitor of cytokinin responses probably by causing competitive inhibition through its binding with the CHKs, RRBs or both [27]. Another example is AXR1 (auxin resistant 1), a key enzyme in the related to ubiquitin (RUB) pathway of protein modification, which promotes the cytokinin response by suppressing the accumulation of the RRA member ARR5 [28]. The GeBP (GL1 enhancer binding protein) and GeBP-like proteins are leucine-zipper transcription factors that promote the cytokinin response by limiting the induction of *RRA* genes [29]. The cytokinin response factors (CRFs) belong to the APETALA2/ethylene responsive factor class of transcription factors and act in parallel to the RRBs in controlling cytokinin response genes [30].

The complexity of the cytokinin signaling pathway is further increased by the existence of multigene families encoding all four core signaling components [15,21,31-34]. Although the current data show that the functional redundancy within these gene families is quite extensive, there is also compelling evidence to suggest some degree of functional diversification [18,19,23,35,36]. To date, two types of functional diversification have been described. First, within all four gene families, members are differentially transcribed both in a tissue- and signal-specific manner, and in terms of relative abundance [37-40]. Second, although proteins within each family share a high degree of identity, their diverged regions are variable enough to offer specific ligand binding affinities or participation in different cellular responses [40-43].

The Arabidopsis RRB family contains 11 members that belong to three phylogenetic groups [19]. All RRBs have a N-terminal receiver domain that includes a conserved aspartate needed for the phosphorelay, a centrally positioned Myb-like DNA binding domain, and a variable domain at the C-terminus which is thought to be responsible for the functional specialization within this family [33,41,43]. Loss-of-function studies with single, double and higher-order mutants have revealed not only a high level of functional redundancy, but also that *ARR1, ARR10, ARR11* and *ARR12* control most of the cytokinin response [23,35,44,45]. Other RRBs are believed to control cytokinin responses in specific tissues or at particular developmental stages. For example, *ARR*2 is predominantly expressed in pollen [46].

Over expression of RRBs leads to cytokinin hypersensitivity, but causes minor changes in plant development [6,18,47]. Based on the analogy with bacterial two-component systems, these observations led to the hypothesis that RRBs are expressed in their inactive forms and that cytokinin promotes the RRBs activation by phosphorylation of a conserved aspartate residue. Indeed, comparative analyses of protoplast expressing wild-type ARR2 and the ARR2^D80N^ loss-of-phosphorylation mutant showed that the cytokinin-dependent induction of the *RRA* gene *ARR6* is reduced in protoplast expressing the ARR2^D80N^ form and that a gel-mobility shift of the ARR2 protein consistent with its phosphorylation is not detectable in the ARR2^D80N^ expressing protoplasts [42]. Studies of two-component signaling systems in bacteria, yeast and plants have shown that a response regulator can be rendered constitutively active if the conserved aspartate is mutated into the phosphomimic residue glutamate [42,48-51]. Indeed, when a *35S:ARR2*^*D80E*^ transgene was expressed in Arabidopsis, plants were dwarfed and their *RRA* genes were constitutively up-regulated [51]. The crucial role of the conserved aspartate for the activation of RRBs was also described in a study of the ARR18 family member [52]. A phosphomimic substitution ARR18^D70E^ also caused a constitutive cytokinin response with respect to the transcriptional induction of primary cytokinin response genes. The effects of phosphomimic ARR18 or ARR2 mutations on cytokinin-regulated developmental and physiological processes were not analyzed [51,52].

Thus, the current data offer little information regarding the effects of overexpressing active, phosphorylated RRBs on intact plants and we still lack final proof that phosphorylation of RRBs is sufficient to promote all the developmental and physiological processes that characterize cytokinin response. To address this issue, we introduced the phosphomimic amino acid substitution D94E in ARR1, one of the major Arabidopsis RRBs, and ectopically expressed the mutant protein in *arr1-1* mutant plants. We show that Arabidopsis seedlings expressing ARR1^D94E^, but not the unmodified ARR1, resemble cytokinin-treated wild-type plants in a transgene dose-dependent manner. Furthermore, our analyses reveal that all of the tested cytokinin responses were constitutively up-regulated in *35S:ARR1*^*D94E*^ plants. Together, our results show that the ARR1^D94E^ protein is a wide-spectrum cytokinin response activator.

## Results and discussion

### The phosphomimic D94E substitution promotes a 100-fold increase in ARR1 activity

**Figure 1:**
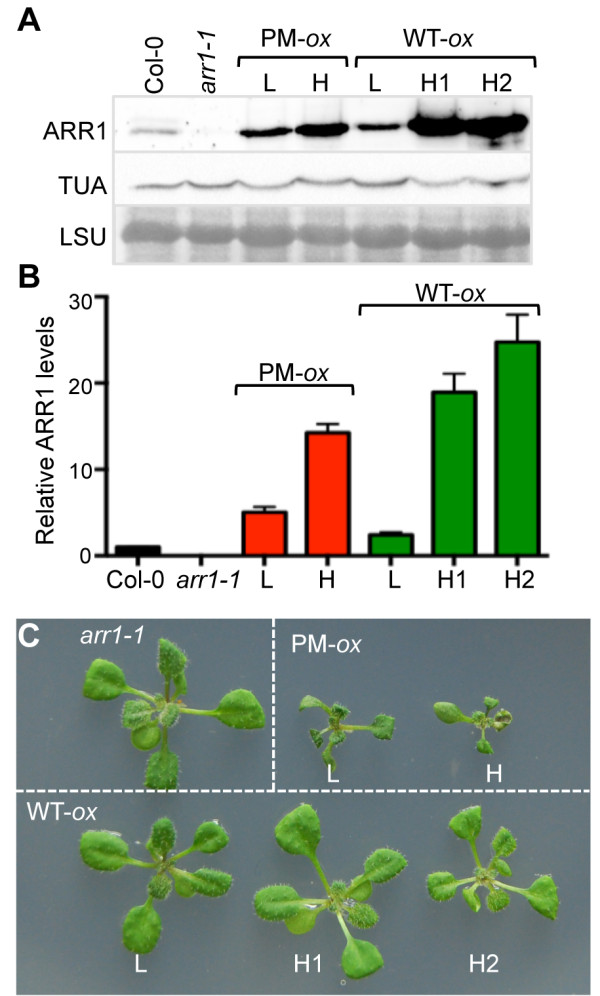
**Analyses of ARR1 expression levels in phosphomimic *35S:ARR1***^***D94E ***^***(*PM-*ox*) and *35S:ARR1 (*WT-*ox*) lines and the characteristic dwarf phenotype of *35S:ARR1***^***D94E***^** plants. (A)** The ARR1 levels in the Col-0 wild type, the *arr1-1* mutant and the overexpression lines generated in the *arr1-1* background were determined using anti-ARR1 antibody. Immunoblotting analysis with the anti-α-tubulin (TUA) antibodies and the image of the region of the Ponceau S-stained membrane surrounding the large RuBisCO subunit (LSU) are shown as controls. L, low and H, high expression levels. **(B)** Quantification of ARR1 expression levels. Chemiluminescent ARR1 signals were normalized to TUA and results are expressed as fold increase compared to Col-0, which was assigned a value of 1. Data are presented as mean ± SEM of two independent experiments. **(C)** Rosettes of representative two-week-old plants grown on MS/2 media.

To compare the capacity of different ARR1 versions to promote cytokinin responses in the absence of cytokinin treatments, we generated plants expressing wild-type ARR1 and the phosphomimic ARR1^D94E^ in the *arr1-1* background (Figure 1). Based on the results of immunoblotting screens of transgenic lines, we selected two *35S:ARR1*^*D94E*^ and three *35S:ARR1* lines for further analyses. The two phosphomimic lines contained ~5 ± 1 and ~14 ± 2 more ARR1 compared to Col-0 plants of the same age, and are here referred to as low (L) and high (H) expressors (Figure 1A, B). The three lines expressing wild-type ARR1 contained ~2.5 ± 0.5, 18 ± 4 and 24 ± 4 more ARR1 compared to Col-0, and are referred to as low (L) and high (H1 and H2) expressing lines, respectively (Figure 1A, B). Despite similar expression levels of phosphomimic and wild-type ARR1 in the H lines, only transgenic plants expressing phosphomimic ARR1 had rosettes smaller than Col-0 and *arr1-1* (Figure 1C). Reduced rosette size is one of the characteristic developmental phenotypes associated with exogenous cytokinin treatments and cytokinin overproduction [18,53].

**Figure 2:**
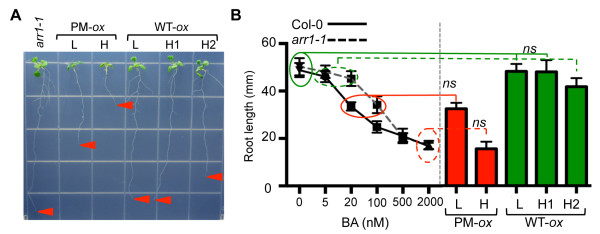
**Quantification of cytokinin response strength in ARR1 overexpressing lines. (A)** Representative ten-day-old *arr1-1, 35S:ARR1* (WT-*ox*) *a*nd *35S:ARR1*^*D94E*^*(*PM-*ox*) seedlings grown on MS/2 medium. The arrows point at the root tips. **(B)** Quantification of root elongation growth. Three-day-old transgenic seedlings grown on MS/2 plates were transferred to fresh MS/2 plates. In parallel, three-day-old Col-0 and *arr1-1* seedlings were transferred MS/2 plates containing the indicated concentrations of BA. The root length of each seedling was marked at the moment of transfer (initial length) and after 7 days of growth on test media (final length). Root elongation (difference between final and initial root length) of 30 seedlings per line is presented as a dose–response curve for Col-0 and *arr1-1* and as bars (mean ± SD) for the transgenic lines. The results were analyzed using one-way analysis of variance (ANOVA) followed by Tukey's multiple comparisons test. The Col-0 and *arr1-1* root lengths that did not significantly (*ns*) differ from the root length of the transgenic line are encircled.

Inhibition of root growth is one of the most sensitive organismal responses to cytokinin [4,54]. Consistent with the effect of the transgene on rosette size, roots of plants expressing phosphomimic ARR1 were shorter than roots of *arr1-1* plants (Figure 2A). We also observed a dose-dependent effect of the phosphomimic ARR1 transgene: roots of the L line were ~2-fold longer than roots of the H seedlings. The root length of plants expressing the wild-type ARR1 was affected only in the strongest expressor line (i.e., H2), and to a lesser extent compared to the phosphomimic ARR1-expressing lines (Figure 2A).

We next estimated the strength of the cytokinin effect caused by the ectopic expression of wild-type and phosphomimic ARR1 versions by comparing the transgenic root lengths with those of the wild-type and *arr1-1* plants grown on MS/2 media supplemented with a range of benzyladenine (BA) doses (Figure 2B). The *35S:ARR1*^*D94E*^ lines showed a constitutive cytokinin response phenotype, with the L and H lines resembling wild-type seedlings treated with 20 nM BA and 0.5 - 2 μM BA, respectively (Figure 2B). In agreement with the observed decrease in cytokinin sensitivity of the *arr1-1* mutant [18], the BA concentration that promoted a similar root growth inhibition was higher (100 nM vs. 20 nM for *arr1-1* and Col-0 respectively; Figure 2B). Analyses of the lines expressing wild-type ARR1 showed that only the highest expressor H2 had a weak constitutive cytokinin response phenotype which resembled wild-type plants grown on 5 nM and *arr1-1* plants grown on 20 nM BA (Figure 1B). To obtain an estimate of the difference in response activation between ARR1 and ARR1^D94E^, we compared the BA doses that phenocopied the root length of the H phosphomimic and the H2 wild-type ARR1 lines. Because the ARR1 level in the wild-type expressor line H2 is higher than in the phosphomimic H line (Figure 1A, B), this comparison represents an estimate of the minimal relative response activation strength of the phosphomimic ARR1^D94E^. Since phosphomimic ARR1 H seedlings resembled the wild type grown on 0.5 μM BA and wild-type ARR1 H2 seedlings resembled the wild type on 5 nM BA, we concluded that the ARR1^D94E^ protein is at least 100-fold more potent in promoting this cytokinin response.

### Ectopic expression of ARR1^D94E^ promotes a wide spectrum of constitutive cytokinin responses

**Figure 3:**
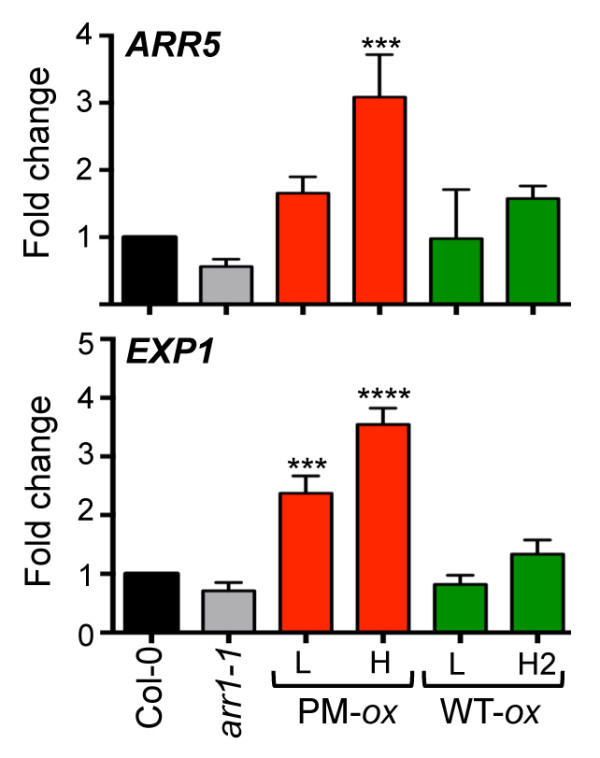
**Analyses of steady-state levels of cytokinin response genes in *35S:ARR1***^***D94*E**^** (PM-*ox*) and *35S:ARR1 *(WT-*ox*) transgenic plants.** The relative transcript levels of the primary response gene *ARR5 (*At3g48100) and the cytokinin-regulated gene *EXPANSIN1* (*EXP1;* At1g69530) in six-day-old seedlings were measured using qRT-PCR. The expression levels were standardized to *GAPDH* (At1g13440) and the value in Col-0 was set to 1. Data are mean ± SD from two biological replicates with three technical replicates each. The results were analyzed using one-way ANOVA followed by Tukey's multiple comparisons test. ***, *P* < 0.001; ****, *P* < 0.0001.

To document the extent of the constitutive response in the phosphomimic lines, we next analyzed other cytokinin-regulated traits in wild-type ARR1 and phosphomimic ARR1 expressing lines. First, we determined the steady-state mRNA levels of two cytokinin-inducible genes. The *RRA* gene *ARR5* is a primary cytokinin response gene and encodes an inhibitor of the cytokinin response [20,25,26]. The cytokinin-inducible *EXP1* gene is thought to act further downstream in the cytokinin response pathway, and it encodes a cell-wall loosening protein expansin 1 [55]*.* Consistent with the constitutive cytokinin response phenotype, the steady-state levels of both *ARR5* and *EXP1* were up-regulated by the phosphomimic *ARR1* transgene (Figure 3). We observed a significant increase in *ARR5* abundance in the phosphomimic ARR1 H line, while the *EXP1* transcript level was higher in both phosphomimic ARR1 lines. In comparison, the expression of both cytokinin-inducible genes was not increased in the wild-type ARR1 expressing lines (Figure 3).

**Figure 4:**
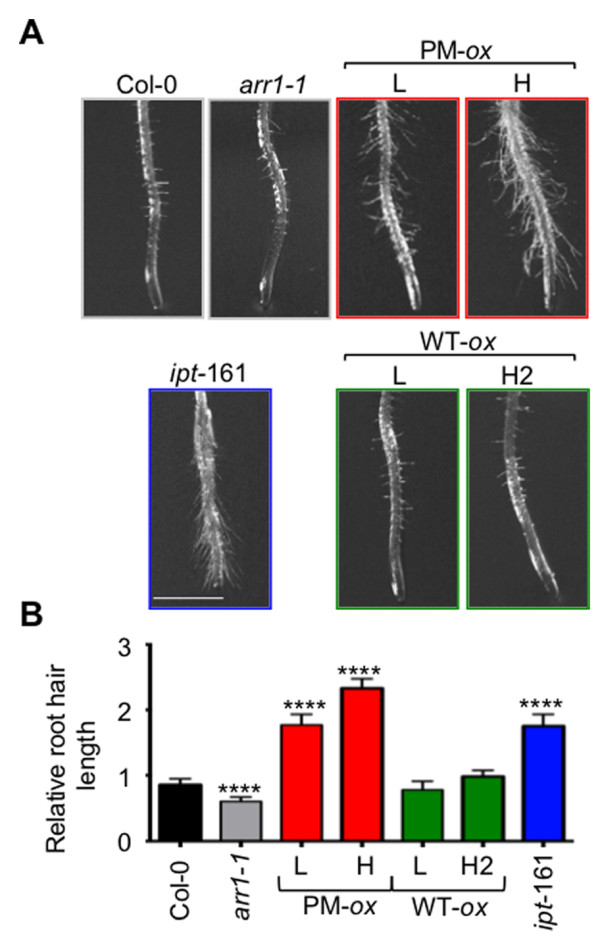
**Effects of phosphomimic ARR1 mutation on root hair length. (A)** Root tips of seedlings grown on vertically positioned MS/2 plates were photographed after five days of growth. The *IPT:ipt* transgenic line *ipt-*161, which has an increased endogenous cytokinin levels [28,53], is included for comparison. Scale bar = 1 mm. **(B**) Root hair lengths in five-day-old plants were measured from micrographs. Six to 12 roots per line were analyzed and at least 10 root hairs per root were measured. Data are presented as mean ± SEM (n ≥ 60). Root hair lengths in different lines were compared to Col-0 using a one-way ANOVA with *post-hoc* Tukey’s multiple comparison test. ****, *P* < 0.0001.

Second, we tested the root hair elongation response which is known to be promoted by exogenous cytokinin [54] and increased in the cytokinin overproducer line *ipt*-161 [28]. Both phosphomimic ARR1 lines had a constitutive root hair elongation response which was transgene dose-dependent (Figure 4). In contrast, no increased root hair elongation was observed in the ARR1 expressing lines. In agreement with the observed decrease in cytokinin sensitivity of the *arr1-1* mutant [18], the root hair length in the *arr1-1* mutant was reduced compared to the wild type (Figure 4).

**Figure 5:**
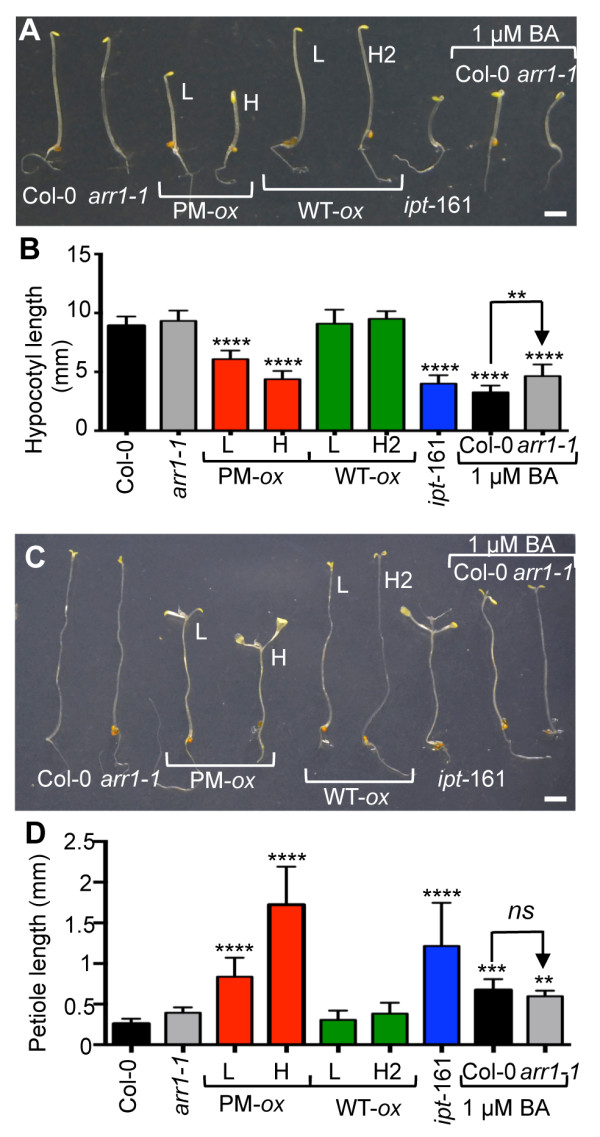
**Constitutive cytokinin responses of etiolated seedlings expressing phosphomimic ARR1. (A)** Four-day-old *35S:ARR1* overexpressing (WT-*ox*) *a*nd *35S:ARR1*^*D94E*^ overexpressing (PM-*ox*) etiolated seedlings are shown. Scale bar = 2 mm. **(B)** Hypocotyl length of four-day-old seedlings was measured from photographs using ImageJ and is presented as mean ± SD (n ≥ 20). Unless marked differently, lines were compared to Col-0 using a one-way ANOVA with Tukey’s multiple comparison test. **, *P* < 0.01 and ****, *P* < 0.0001. **(C)** Four-week-old etiolated seedlings. Scale bar = 2 mm. **(D)** The length of the cotyledonary petioles was measured from photographs using ImageJ and presented as mean ± SD (n ≥ 12). **, *P* < 0.01, ***, *P* < 0.001 and ****, *P* < 0.0001 for one-way ANOVA with Tukey’s multiple comparison test. *ns,* not statistically significant (*P* > 0.05).

Next we tested the cytokinin responses of transgenic lines grown in darkness. Previous studies have identified two types of cytokinin growth responses in etiolated seedlings [56]. At an early stage of etiolated growth (3 to 5 days of dark incubation), cytokinin-treated seedlings have a shorter hypocotyl and an increased apical hook curvature, whereas at later stages (e.g., after 4 weeks), cytokinin-treated seedlings have swollen upper hypocotyls regions, expanded cotyledons and have developed true leaves [56,57]. The untreated wild type and *arr1-1* seedlings did not differ from each other and displayed the expected etiolated development that is characterized by an elongated hypocotyl and small yellow unopened cotyledons (Figure 5A). In contrast, the *35S:ARR1*^*D94E*^ plants again exhibited a constitutive cytokinin-response phenotype when grown in darkness (Figure 5). After 4 days of growth in the dark, both *35S:ARR1*^*D94E*^ lines had shorter hypocotyls and an exaggerated apical hook curvature, and resembled the cytokinin-treated wild-type and *ipt*-161 seedlings (Figure 5A, B). After 4 weeks of growth in the dark, both the *35S:ARR1*^*D94E*^ and *ipt*-161 seedlings had expanded cotyledons, swollen upper hypocotyls regions and had formed true leaves, again resembling the cytokinin-treated wild type (Figure 5C, D). Contrary to the effect of exogenous cytokinin on the wild type, we did not observe a reduction in root elongation in any of the etiolated seedlings, including the cytokinin overproducer *ipt*-161 (Figure 5A). The lines expressing the wild-type ARR1 did not display any constitutive response phenotype either at the early or late etiolated development stages (Figure 5).

**Figure 6:**
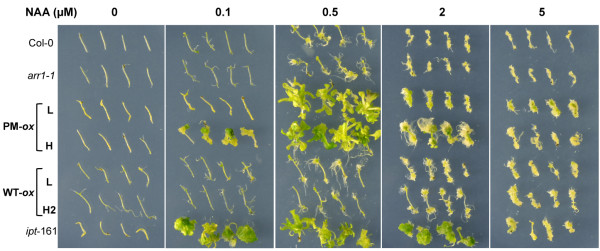
**The response of hypocotyl explants to auxin.** Hypocotyls from Col-0 wild type, *arr1-1* mutant, low (L) and high (H) expressor of phosphomimic (PM) and wild-type (WT) ARR1, and cytokinin overexpressor *ipt*-161 were excised from six-day-old seedlings, transferred to media containing the indicated doses of the auxin 1-naphthaleneacetic acid (NAA) and photographed after 28 days of treatment. Four hypocotyls per treatment are shown for each line.

The fourth test we conducted is the analyses of hypocotyl and root explants responses to auxin (Figure 6). Incubation of explants on media supplemented with particular concentration ratios of auxin and cytokinin are known to promote callus or shoot formation [58]. Typically, a high cytokinin-to-auxin ratio will promote the formation of green calli and the occasional shoot development. In contrast, a lower cytokinin-to-auxin ratio will promote the growth of only white calli or if the ratio drops below a critical threshold, no cell proliferation at all. In theory, a constitutive cytokinin response mutant or a cytokinin overproducer would not require cytokinin in the media for callus or shoot formation. Indeed, after 28 days of incubation on media containing only 0.1 μM NAA, hypocotyls excised from *35S:ARR1*^*D94E*^(H) and *ipt-*161 seedlings developed green calli (Figure 6). Higher concentration of NAA (0.5 μM) promoted green callus and shoot formation in both phosphomimic lines and in *ipt-*161 (Figure 6). As expected, no cell proliferation was observed in wild-type and *arr1-1* hypocotyls on any of the auxin concentrations tested. We also did not observe any callus or shoot induction responses in the wild-type ARR1 overexpressing lines confirming that these lines do not have a constitutive cytokinin response (Figure 6). Therefore, the cell proliferation response to auxin required either an increase in endogenous cytokinin or a constitutive up-regulation of ARR1 action. Because the cell proliferation and differentiation was more pronounced in the strong *35S:ARR1*^*D94E*^ compared to the weak *35S:ARR1*^*D94E*^ line, we concluded that the relative response strengths were a reflection of the difference in ARR1^D94E^ expression level. It was shown earlier that overexpression of the unmodified ARR1 form promotes a hypersensitive cytokinin response in tissue culture and leads to the formation of green callus even in the absence of exogenous cytokinin [18]. By comparing the effects of similarly expressed wild-type and phosphomimic ARR1, we show that ARR1^D94E^ is much more potent at promoting this type of cytokinin-independent tissue culture response.

**Figure 7:**
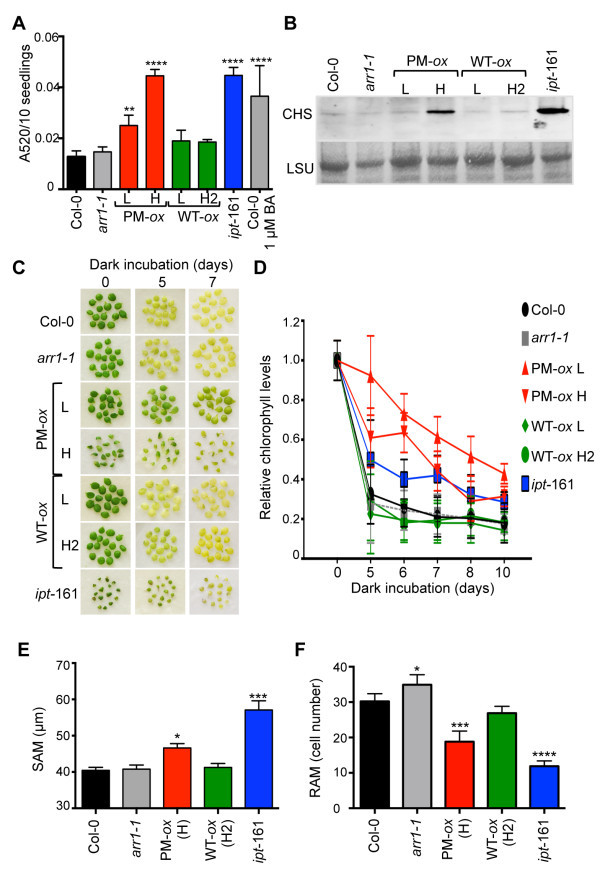
**Constitutive cytokinin responses of *35S:ARR1***^***D94***^** (PM-*ox*) lines in anthocyanin accumulation, the timing of senescence and meristem size. (A)** The anthocyanin content, expressed as absorbance value at 520 nm per 10 seedlings, was measured in six-day-old seedlings. The mean ± SD of three biological replicates is shown. Col-0 was treated with BA for 16 hours. One-way ANOVA with Tukey’s test was used to analyze the data. **, *P* < 0.01; ****, *P* < 0.0001. **(B)** Immunoblotting analyses of chalcone synthase (CHS) levels in eight-day-old seedlings. **(C)** Delayed senescence in PM-*ox* plants. Seedlings of the wild type, *arr1-1,* PM-*ox*, WT-*ox* and *ipt*-161 lines were grown on MS/2 media for 5 days, and then used to dissect cotyledons. Cotyledons were placed on moistened filter papers in petri dishes, and transferred to darkness. Photographs were taken at the indicated times. **(D)** Relative chlorophyll levels in cotyledons incubated in the dark for the indicated times. Chlorophyll content at day one for each line was assigned the value of 1. Mean ± SD of two independent samples (with 15 cotyledons each) is shown. **(E)** Analyses of the shoot apical meristem (SAM) size in six-day-old seedlings. SAM diameters measured from photomicrographs are presented as mean ± SEM (*n* = 25). Results were compared to Col-0 using a one-way ANOVA with Tukey’s test.*, *P* < 0.05; ***, *P* < 0.001. **(F)** Size of the root apical meristem (RAM) was analyzed in seven-day-old seedlings grown on vertically positioned plates. The number of RAM cortex cells measured from photomicrographs is presented as mean ± SEM (*n* = 25). Significance of the difference with Col-0 is shown. *, *P* < 0.05; **, *P* < 0.01 (one-way ANOVA with Tukey’s test).

Cytokinin treatments have been shown to increase the transcript levels of a number of genes encoding flavonoid biosynthetic enzymes [59]. Some of the flavonoid biosynthetic genes (e.g., chalcone synthase (*CHS*) gene encoding the key enzyme of the flavonoid biosynthesis pathway CHS [60,61] or dihydroflavonol reductase genes) were transcriptionally induced by BA, whereas the steady-state mRNA levels of others (e.g., chalcone isomerase) were increased via a post-transcriptional mechanism [59]. In agreement with the described effects of cytokinins on anthocyanin production, we found that anthocyanins accumulated to a higher level in *35S:ARR1*^*D94E*^ seedlings compared to the *35S:ARR1* lines, wild type and the *arr1-1* mutant, which contained statistically identical amounts of anthocyanins (Figure 7A). These increases were approximately of the same magnitude as those measured in *ipt*-161 seedlings or in Col-0 treated with 1 μM BA. The *35S:ARR1*^*D94E*^(H) plants accumulated more anthocyanins than the *35S:ARR1*^*D94E*^(L) plants, confirming that the transgene promotes cytokinin responses in a dose-dependent manner. The CHS level was increased only in the *35S:ARR1*^*D94E*^ (H) and *ipt*-161 seedlings which was in agreement with the high anthocyanin content of these lines (Figure 7A, B).

Cytokinins are known to inhibit leaf senescence and its hallmark symptom, chlorophyll breakdown [62-64]. It has been reported that artificially-induced chlorophyll loss caused by the incubation of detached leaves in darkness and senescence-induced chlorophyll loss are both mediated by the same mechanism [65]. To determine senescence progression under controlled conditions, we performed detached-leaf senescence tests and used cotyledons that are developmentally of the same age in the wild type and transgenic lines. We observed a significant senescence delay in both the *35S:ARR1*^*D94E*^ and *ipt*-161 lines when compared to the wild type and *arr1-1* mutant (Figure 7C, D). For all three lines, the senescence delay was especially clear from days 5 to 7 into the treatment (Figure 7C, D). However, for this cytokinin-response, we observed no correlation between the strength of the cytokinin phenotype and the ARR1^D94E^ expression level: in contrast to the other analyzed cytokinin responses where the *35S:ARR1*^*D94E*^ (L) line consistently had the weaker cytokinin phenotype, the *35S:ARR1*^*D94E*^ (L) line showed the strongest senescence delay (Figure 7C, D). No changes in senescence-induced chlorophyll loss were observed in any of the wild-type ARR1 overexpressing lines or in the *arr1-1* mutant.

Finally, it has been shown that cytokinins have a role in the regulation of meristem development [66]. Decrease in cytokinin sensitivity or content causes a decrease in shoot apical meristem (SAM) size, whereas increased cytokinin action promotes a SAM size increase [31,32,67-70]. Analyses of six-day-old seedlings revealed a significant SAM size increase in the *35S:ARR1*^*D94E*^ (H) line and in *ipt-*161 seedlings (Figure 7E). No changes of the SAM size were detected in seedlings overexpressing wild-type ARR1 or in the *arr1-1* mutant (Figure 7E). On the other hand, a decrease in cytokinin sensitivity or content was shown previously to promote an increase in root apical meristem (RAM) size, whereas increased cytokinin action promoted a RAM size decrease [71-73]. Consistent with their increased cytokinin action, we observed a decrease in RAM size in both *35S:ARR1*^*D94E*^ lines, while the ARR1 overexpressing lines did not significantly differ from the wild type (Figure 7F). In agreement with the previous reports [72], we observed an increase in RAM size in the *arr1-1* seedlings (Figure 7F). Collectively, these analyses confirmed the constitutive cytokinin response phenotype of the *35S:ARR1*^*D94E*^ lines.

### Ethylene-dependent constitutive cytokinin responses in 35S:ARR1^D94E^ overexpressing lines

**Figure 8:**
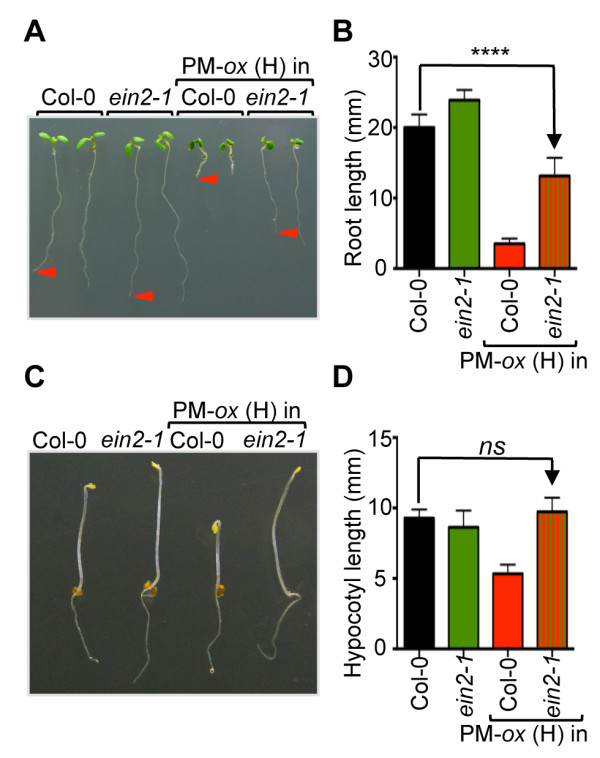
**Ethylene-dependent constitutive cytokinin responses in *35S:ARR1***^***D94E***^** (PM-*ox*) seedlings. (A)** Representative seven-day-old Col-0, *ein2-1,* PM-*ox* (H) and PM-*ox* (H) *ein2-1* plants grown on vertical MS/2 plates. **(B)** Root length of seven-day-old seedlings was measured from photographs. Data are presented as mean ± SD (*n* = 20). One-way ANOVA with Tukey's multiple comparison test was used to analyze the statistical significance of the results. Only the significance of the difference between Col-0 and PM-*ox* (H) *ein2-1* is shown. ****, *P* < 0.0001. **(C)** Representative four-day-old seedlings germinated and grown in darkness on MS/2 medium. **(D)** Hypocotyl length of four-day-old etiolated seedlings. Data are presented as mean ± SD (*n* = 20). Only the significance of the difference between Col-0 and PM-*ox* (H) *ein2-1* is shown. *ns*, not significant (one-way ANOVA with Tukey's multiple comparison test).

Some of the effects of cytokinin on plant development are mediated by increased ethylene biosynthesis that is caused by the stabilization and thus increased activity of the key ethylene biosynthesis enzyme ACC synthase [56,74,75]. In dark-grown seedlings, cytokinin promotes a triple response that includes an increase in apical hook curvature and an inhibition in both hypocotyl and root elongation [56]. In light-grown seedlings, the inhibitory effect of cytokinin on root elongation is also in part mediated by increased ethylene production [74]. To test if the reduced hypocotyl elongation in dark-grown *35S:ARR1*^*D94E*^ seedlings is a result of increased ethylene action, we introgressed the *35S:ARR1*^*D94E*^ (H) line into the ethylene insensitive mutant *ein2-1* that carries a defect in a key step of the ethylene response pathway [76]. In light-grown seedlings, loss of EIN2 function partially suppressed the short-root phenotype of the *35S:ARR1*^*D94E*^(H) seedlings (Figure 8A, B). In contrast, *ein2-1* completely suppressed the short-hypocotyl phenotype of etiolated *35S:ARR1*^*D94E*^(H) seedlings (Figure 8C, D). A significant portion of the inhibitory effect of cytokinins on the root and hypocotyl elongation growth of young seedlings is mediated through an increase in ethylene action caused by a cytokinin-induced increase in ethylene biosynthesis [75]. It was shown earlier that this cytokinin-induced ethylene biosynthesis involves the primary cytokinin response pathway [77]. Our results confirm this order of events by showing that the inhibitory effects of increased ARR1 action on elongation growth is suppressed in an ethylene insensitive background. However, whereas the suppression of hypocotyl growth inhibition was complete in etiolated seedlings, we observed only a partial reversion of root elongation growth in light-grown seedlings. Considering that the *ein2-1* mutation causes a near complete ethylene resistance [78], it is unlikely that the partial reversion in light-grown seedlings is caused by residual ethylene action. Instead, cytokinin action probably controls root elongation via an ethylene-independent pathway. Indeed, it was shown earlier that cytokinin regulates root growth by controlling the distribution of auxin that impacts the activity and size of the root meristem [79].

Our results with *35S:ARR1*^*D94E*^ stand in contrast to results obtained with plants expressing *35S:ARR2*^*804E*^, the phosphomimic version of ARR2 [51]. In this earlier study, it was concluded that ARR2 plays a role in the ethylene response pathway because the constitutive triple response phenotype of seedlings expressing *35S:ARR2*^*804E*^ was not suppressed by ethylene biosynthesis inhibition [51]. In contrast, our observation that the etiolated phenotype of *35S:ARR1*^*D94E*^ seedlings is fully suppressed by loss of function of the EIN2 ethylene signaling component (Figure 8), indicated that the constitutive triple response of *35S:ARR1*^*D94E*^ seedlings is caused by an increase in ethylene biosynthesis which is in agreement with the well-established stimulatory role of cytokinin on ACC synthase activity [56,74,75]. Taken together, this may be yet another example suggesting that ARR2 has a specific function in comparison to other RRBs such as ARR1 [41,42,51,80,81].

### Developmental changes in adult 35S:ARR1^D94E^ plants

**Figure 9:**
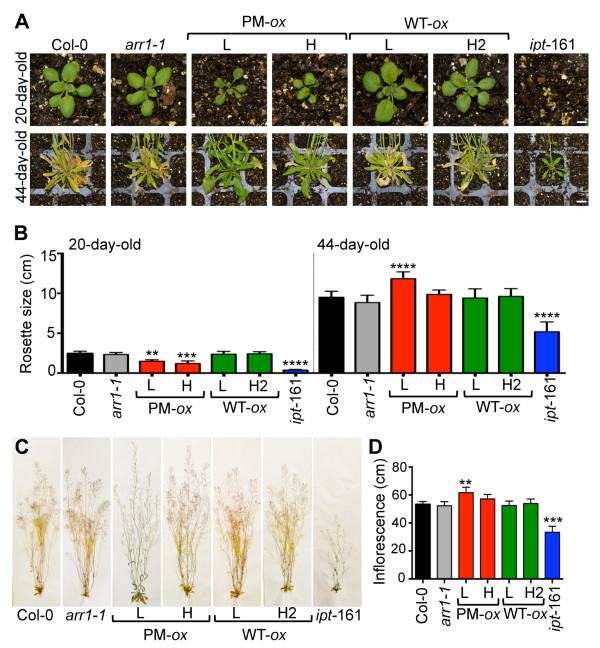
**Rosette and inflorescence development in *35S:ARR1***^***D94E***^** lines. (A)** Representative 20- and 44-day-old soil-grown plants. Scale bars = 0.5 cm (upper panels), 2 cm (lower panels). **(B)** Rosette diameter of ≥12 plants per line was measured and is presented as mean ± SD. The significance of the difference between Col-0 and transgenic lines is shown. **, *P* < 0.01; ***, *P* < 0.001; ****, *P* < 0.0001 (one-way ANOVA with Tukey's multiple comparison test). **(C)** Inflorescences of the wild-type, *35S:ARR1*^*D94E*^ (PM-*ox*) and *35S:ARR1* (WT-*ox*) lines and *ipt*-161 after prolonged growth (44 days) on soil. Photographs of dry, herbarium specimens are shown. **(D)** Main inflorescence length of plants shown in C. Data are presented as mean ± SD (*n* = 12). Only the significance of the difference with Col-0 is marked. **, *P* < 0.01; ***, *P* < 0.001 (one-way ANOVA with Tukey's multiple comparison test).

Both the *35S:ARR1*^*D94E*^ and *ipt*-161 plants remained smaller than the wild type during the early stages of adult development (Figure 9A, B). However, after 44 days of growth, the rosette size of the *35S:ARR1*^*D94E*^ (L) plants was increased compared to the wild type and *arr1-1* (Figure 9A, B). These larger rosettes were also visibly greener which was in agreement with the senescence delay observed with the cotyledon assay (Figure 7C, D). The ARR1 overexpressing lines and *arr1-1* mutant had wild-type sized rosettes. The *ipt*-161 plants, which required a longer growth period to reach their final size, remained substantially smaller after prolonged growth on soil (Figure 9A, B).

Cytokinins are known to be involved in the regulation of shoot branching. Although the hormonal regulation of shoot branching has been discovered many decades ago, new hormones that influence this process and the identities of the effectors involved are still being discovered [82-86]. Because cytokinins increase the size of shoot apical meristems, and because they are known to promote the release of apical dominance when directly applied to lateral buds, it is commonly believed that cytokinins increase shoot branching [67,82,87]. However, the inflorescences of *35S:ARR1*^*D94E*^ plants were not visibly more branched than inflorescences of the wild type or *arr1-1* (Figure 9C). The length of the primary inflorescence stem was significantly increased in the *35S:ARR1*^*D94E*^ (L) line (Figure 9C, D), which is a likely consequence of the delayed senescence phenotype observed in these plants (Figures 7C, D and 9A, B). The inflorescence of *ipt-*161 plants was substantially shorter and not more branched compared all other lines (Figure 9C, D), which is in agreement with what was reported earlier for the C24 ecotype version of this transgenic line [53]. One unexpected feature of the *35S:ARR1*^*D94E*^ inflorescence phenotype was the absence of any visible increase in branching accompanied by a loss in shoot apical dominance. Considering the classical role of cytokinins in promoting release of apical dominance [5,67,82,87], one would expect that increased cytokinin action would lead to a bushier inflorescence structure. However, recent studies have shown that the down-regulation of cytokinin biosynthesis and suppression of RRBs function increase inflorescence branching [88,89]. These studies combined with our data suggest that cytokinins play a more complex role in this developmental process. However, it also remains possible that the effect of the *35S:ARR1*^*D94E*^ transgene is suppressed at this later developmental stage and that we therefore did not observe any effects on inflorescence development.

## Conclusions

Here we show that seedlings ectopically expressing a phosphomimic version of ARR1 resembled the cytokinin-treated wild type, and that the relative strengths of most of the cytokinin-related phenotypes correlated with ARR1^D94E^ abundance. Furthermore, we showed that the constitutive cytokinin response phenotype, which was not observed in ARR1 overexpressing plants, is the result of a significant increase in the capacity to promote cytokinin responses in the absence of exogenous cytokinin application.

Because we used the constitutive CaMV 35S promoter, our results do not interpret the function of the *ARR1* gene in Arabidopsis development. Rather, they enable us to reach two important conclusions about the ARR1 protein. First, because the phosphomimic substitution constitutively activated the cytokinin response both at the molecular, physiological and developmental levels, we concluded that ARR1 phosphorylation at D94 is indeed a key step in cytokinin signaling. The D94E substitution converted ARR1 from a latent into an active transcription factor which is 100-fold more potent as a response activator compared to its wild-type counterpart. The second conclusion is that phosphomimic (and presumably phosphorylated) ARR1 has the capacity to promote most of the currently known cytokinin responses. However, it remains possible that ARR1^D94E^ does not promote cytokinin responses that were not analyzed in this study.

Our results show that the *35S:ARR1*^*D94E*^ transgene mimics the effects of cytokinin treatments, and hence, validate the current model of cytokinin signaling which stresses the essential role for phosphorylation of RRBs on their conserved aspartate residue in promoting a wide-spectrum of cytokinin responses. Whereas our results provide information about the ARR1 protein and the cytokinin response pathway in general, the observation that a phosphomimic version of ARR1 can be used as a wide-spectrum cytokinin response activator is also relevant for biotechnology and agriculture. Cytokinins regulate a number of developmental processes and environmental responses that are of significance for crop yields [90,91]. So far, the engineering of cytokinin-controlled agriculturally important traits has focused predominantly on modifying cytokinin accumulation either via changes in biosynthesis or metabolism [90]. An alternative approach could be the use of constitutively active signaling proteins in combination with tissue or developmental stage specific promoters. Based on the high level of conservation of the cytokinin response pathway in higher plant species [92], it is reasonable to assume that phosphomimic versions of the corresponding ARR1 versions of crop species will be useful for the engineering of cytokinin-related traits.

## Methods

### Plant material and growth conditions

For all experiments, *Arabidopsis thaliana* wild-type, transgenic and mutant plants (all in Col-0 background) were germinated and grown under sterile conditions. Surface-sterilized seeds were stratified for two days and plated on half-strength Murashige and Skoog medium (MS/2; 0.5x Murashige and Skoog salts with 1% sucrose, pH 5.7). Plants were grown in a growth chamber at 22°C under a 16 hr light (80 μmol m^-2^ s^-1^)/8 hr dark cycle. For growth on soil, sterile seedlings were transferred to a 1:1 mix of Potting Mix soil (Fertilome) and Vermiculite Perlite (Therm-o-Rock East Inc.). The *arr1-1* and *ein2-1* mutants and the *IPT:ipt* transgenic line *ipt-*161 in Col-0 background were described previously [18,28,78].

To generate wild-type and phosphomimic ARR1 overexpressing plants, the *ARR1* (*ARR1;* At3g16857) cDNA was PCR-amplified from an Arabidopsis cDNA library using attB primers, and cloned into pDONR221 using BP clonase enzyme mix (Invitrogen). The resulting pENTR-*ARR1* clone was used for site-directed mutagenesis with forward and reverse primers 5’-GATGTTCAT ATGCCTGAGATGGACGGTTTCAAG-3’ that introduce the C-to-G mutation, and thus D to E substitution at position 94. The wild-type and the *ARR1*^D94E^ fragment were recombined into the pEarlyGate100 binary vector [93] using LR clonase enzyme mix (Invitrogen). The construct were introduced into the C58C1Rif *Agrobacterium tumefaciens* strain which was used for floral dip transformation [94]. Transgenic plants were selected on solid MS/2 medium containing 1% sucrose and 10 μg/ml phosphinothricin (GoldBio).

### Protein isolation and immunoblotting analyses

For immunoblotting, total proteins were isolated in 2X SDS-PAGE loading buffer, separated by SDS-PAGE, and transferred to nitrocellulose membranes as described [95]. Commercial antibodies used were monoclonal anti-α tubulin antibody (dilution 1:10,000; clone B-5-1-2, Sigma) and anti-chalcone synthase antibody (dilution 1:1000; Santa Cruz Biotechnology). To generate ARR1-specific antibodies, a 55 amino acid-long peptide (amino acids 348 to 402) was chosen as an antigen. This peptide has 47% amino acid sequence identity with ARR1 homologue ARR2. The longest consecutive stretch of identical amino acids in this region of ARR1 and ARR2 is six. The antisera were generated in rabbits, and affinity purified against the antigen before use (Strategic Diagnostics). The affinity-purified antiserum was used after 1:10,000 dilution. Signals were captured using ChemiDoc XRS, and the signal intensity was determined using Quantity One software (Bio-Rad).

### Cytokinin response assays

#### Inhibition of root elongation

Vertically grown seedlings were transferred to control MS/2 plates and MS/2 plates containing benzyladenine (BA), and the initial root length was marked. Seedlings were grown vertically for an additional 7 days. Root lengths were measured using ImageJ (http://rsb.info.nih.gov/ij/).

#### Induction of cytokinin-responsive genes

RNA was isolated using the TRIzol reagent (Invitrogen) from plants grown in liquid MS/2 media for 7 days. The qRT-PCR analyses and the sequence of *ARR5* primers was described [28]. Primers used for the analyses of *EXP1* levels were 5’-CAACGCATCGCTCAATACAG-3’ and 5’-CTCCGACGTTAGTGATCAGAAC-3’.

#### Callus and shoot induction in root and hypocotyls explants

Hypocotyls of plants grown in darkness for four days and then in light for two days were excised and transferred to full-strength MS media supplemented with 2% sucrose and naphthalene-1-acetic acid (NAA). A minimum of 40 hypocotyls per line was tested for each NAA concentration. Test plates were kept in a controlled environment chamber with continuous light and temperature of 22°C, and were followed daily.

#### Anthocyanin accumulation

Seedlings grown for six days on MS/2 media were collected (10 per sample), submerged into 500 μl of acid methanol (1% HCl), and rocked at 4°C for 12 hours in darkness. The anthocyanin fraction was extracted using chloroform phase separation as described [60]. The anthocyanin content was measured a DTX 880 Multimode Detector (Beckman Coulter) with a 520/8 nm absorbance filter.

#### Cotyledon senescence

Cotyledons of five-day-old light-grown seedlings were excised and transferred to Petri dishes with a filter paper moistened with distilled water. Samples were incubated in the dark. At the denoted time intervals, cotyledons were photographed and a minimum of 15 cotyledons per line was frozen in liquid nitrogen for chlorophyll extraction. For chlorophyll extraction, frozen cotyledons were incubated with 80% (v/v) acetone at 4°C for 12 hours in the darkness. Absorbance at 647 and 664 nm was measured using Ultrospec 2000 (Pharmacia), and the chlorophyll amount was calculated according to Graan and Ort [96].

#### SAM size measurements

Shoot apical meristem size was analyzed as described [97]. Briefly, six-day-old seedlings were cleared with Hoyer’s solution for 24 to 48 hours. Slides were observed with the Zeiss Axioplan2 and Axiovision software using the 40× objective/1× optivar.

#### Root meristem cell number measurements

Root meristem size was analyzed as described [98]. Briefly, seven-day-old seedlings grown on vertical plates were cleared with Hoyer’s solution for 12 hours, mounted on slides using Hoyer’s solution and observed with the Olympus BX51 microscope (40× objective) equipped with a differential interference contrast technology and a DP70 digital camera.

#### Biometrics

The descriptive statistics, plotting, and statistic analyses were done using Prism 6 (GraphPad). The statistical tests used to analyze the data, the size of tested sample sets and number of biological replicates are stated in the Result and Discussion section or Figure legends.

## Competing interests

The authors declare that there are no competing financial interests.

## Authors’ contributions

JK and JAS devised the experimental setup, performed most of the experiments and analyses, and wrote the manuscript. YL conducted the qPCR analyses, analyzed the anthocyanin and CHS levels. SEP analyzed the SAM size. YL and SEP participated in manuscript editing. All authors have approved the manuscript.
